# A randomized controlled trial comparing perioperative vs. postoperative mFOLFOX6 for lower rectal cancer with suspected lateral pelvic lymph node metastasis (JCOG1310): a phase II/III randomized controlled trial

**DOI:** 10.1093/jjco/hyac080

**Published:** 2022-05-30

**Authors:** Masayuki Ohue, Satoru Iwasa, Junki Mizusawa, Yukihide Kanemitsu, Manabu Shiozawa, Yusuke Nishizawa, Hideki Ueno, Kenji Katsumata, Masayoshi Yasui, Shunsuke Tsukamoto, Hiroshi Katayama, Haruhiko Fukuda, Yasuhiro Shimada

**Affiliations:** Department of Gastroenterological Surgery, Osaka International Cancer Institute, Osaka, Japan; Gastrointestinal Medical Oncology Division, National Cancer Center Hospital, Tokyo, Japan; Japan Clinical Oncology Group Data Center/Operations Office, National Cancer Center Hospital, Tokyo, Japan; Department of Colorectal Surgery, National Cancer Center Hospital, Tokyo, Japan; Department of Surgery, Kanagawa Cancer Center, Yokohama, Japan; Department of Surgery, Saitama Prefecture Cancer Center, Saitama, Japan; Department of Surgery, National Defense Medical College, Saitama, Japan; Department of Gastrointestinal and Pediatric Surgery, Tokyo Medical University, Tokyo, Japan; Department of Gastroenterological Surgery, Osaka International Cancer Institute, Osaka, Japan; Department of Colorectal Surgery, National Cancer Center Hospital, Tokyo, Japan; Japan Clinical Oncology Group Data Center/Operations Office, National Cancer Center Hospital, Tokyo, Japan; Japan Clinical Oncology Group Data Center/Operations Office, National Cancer Center Hospital, Tokyo, Japan; Clinical Oncology Division, Kochi Health Sciences Center, Kochi, Japan

**Keywords:** rectal cancer, lateral pelvic lymph node metastasis, lateral lymph node dissection, perioperative chemotherapy, postoperative chemotherapy

## Abstract

**Objective:**

The optimal perioperative chemotherapy for lower rectal cancer with lateral pelvic lymph node metastasis remains unclear. We evaluated the efficacy and safety of perioperative mFOLFOX6 in comparison with postoperative mFOLFOX6 for rectal cancer patients undergoing total mesorectal excision with lateral lymph node dissection.

**Methods:**

We conducted an open label randomized phase II/III trial in 18 Japanese institutions. We enrolled patients with histologically proven lower rectal adenocarcinoma with clinical pelvic lateral lymph node metastasis who were randomly assigned (1:1) to receive postoperative mFOLFOX6 (12 courses of intravenous oxaliplatin [85 mg/m^2^] with L-leucovorin [200 mg/m^2^] followed by 5-fluorouracil [400 mg/m^2^, bolus and 2400 mg/m^2^, continuous infusion, repeated every 2 weeks]) or perioperative mFOLFOX6 (six courses each preoperatively and postoperatively). The primary endpoint was overall survival (OS). The trial is registered with Japan Registry of Clinical Trials, number jRCTs031180230.

**Results:**

Between May 2015, and May 2019, 48 patients were randomized to the postoperative arm (*n* = 26) and the perioperative arm (*n* = 22). The trial was terminated prematurely due to poor accrual. The 3-year OS in the postoperative and perioperative groups were 66.1 and 84.4%, respectively (HR 0.58, 95% CI [0.14–2.45], one-sided *P* = 0.23). The pathological complete response rate in the perioperative group was 9.1%. Grade 3 postoperative surgical complications were more frequently observed in the perioperative arm (50.0 vs. 12.0%). One treatment-related death due to sepsis from pelvic infection occurred in the postoperative group.

**Conclusions:**

Perioperative mFOLFOX6 may be an insufficient treatment to improve survival of lower rectal cancer with lateral pelvic lymph node metastasis.

## Teaser

This study investigated the efficacy and safety of perioperative mFOLFOX6 in comparison to postoperative mFOLFOX6 for lower rectal cancer patients undergoing total mesorectal excision with lateral lymph node dissection.

## Introduction

Preoperative chemoradiation (CRT) followed by total mesorectal excision (TME) is the standard procedure for the treatment of locally advanced rectal cancer (LARC) in Europe and North America (1). In Japan, however, TME with lateral lymph node dissection (LLND) followed by 5-FU and L-leucovorine is still the standard treatment for rectal cancer without lateral pelvic lymph node metastasis (LLNM), where preoperative chemoradiotherapy is not routinely performed based on the results of JCOG0212 (2–4).

In contrast, rectal cancer with LLNM is well known to be associated with high risk of a worse prognosis, with 5-year OS <40% (5). TME with LLND should be performed with R0 resection to obtain better prognosis in patients of rectal cancer with LLNM (6). TME with LLND followed by adjuvant chemotherapy with mFOLFOX6, which is more intensive than the standard treatment in the JCOG0212, is a standard treatment for rectal cancer with LLNM (7–9). However, due to poor prognosis, the development of new treatments to improve survival in such high-risk patients is an urgent task.

Regarding adjuvant treatment with R0 surgery, several papers reported that CRT with TME did not improve OS of rectal cancer patients and was not efficient for the treatment of LLNM (10,11). Moreover, CRT was associated with acute side effects, postoperative morbidity and late toxicity (e.g. fecal incontinence, anal blood loss, and anal mucus loss) (12). Recently, several small trials of preoperative chemotherapy without radiation reported promising results for low-risk rectal cancer (13,14). We hypothesized that the preoperative introduction of intensive chemotherapy might ameliorate compliance with the protocol, helping to prevent dissemination of micrometastasis in comparison with postoperative chemotherapy alone, and improve survival of high-risk rectal cancer with LLNM. We therefore designed this JCOG1310 trial (UMIN Clinical Trials Registry: UMIN000017603, and Japan Registry of Clinical Trials: jRCTs031180230) to confirm the superiority—in terms of OS—of perioperative (preoperative and postoperative) chemotherapy to postoperative chemotherapy.

## Patients and methods

### Eligibility criteria

**Figure 1:**
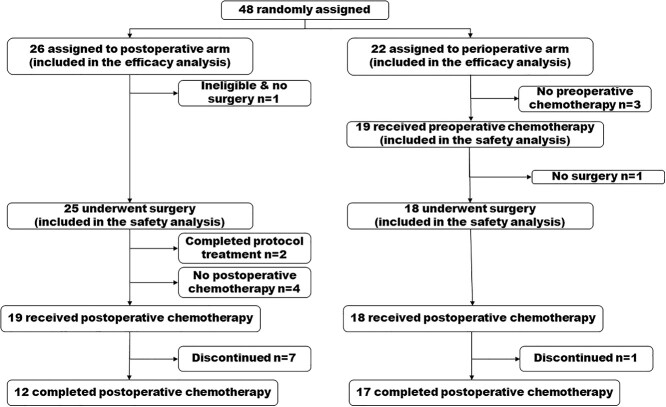
Trial profile.

JCOG1310 was a multicenter, open-label, randomized, phase II/III trial. The study design has been reported in detail elsewhere (9). Rectal carcinoma was classified according to the seventh edition of TNM classification (15) and the eighth edition of the Japanese Classification of Colon and Rectal Carcinoma (16). Eligibility criteria included histologically proven rectal adenocarcinoma, main lesion located in the rectum with the lower margin below the peritoneal reflection, lateral pelvic lymph nodes with a short axis diameter of ≥10 mm on MRI or CT that were cN3 in the Japanese Classification (16), cT2 to cT4 (excluding cT4 tumors invading the trigone of the bladder, urethra, or sacrum), no distant metastasis (cM0), Eastern Cooperative Oncology Group performance status 0 or 1, age 20–74 years, no prior chemotherapy or treatment such as rectal resection, pelvic lymph node dissection, or pelvic irradiation for any malignancies, and no other colorectal carcinoma except cTis or cT1a. After confirming their eligibility, patients were randomized (1:1) to the postoperative chemotherapy and perioperative chemotherapy arms (Fig. 1). The minimization method was used for randomization, with study arms balanced according to sex, tumor depth (T2-3 vs. T4) and institution. The study protocol was approved by the JCOG Protocol Review Committee and the institutional review board of each participating hospital before the initiation of the study. The Data and Safety Monitoring Committee monitored the data and operation of the study. This study was conducted in accordance with the international ethical recommendations of the Declaration of Helsinki, the Ethical Guidelines for Medical and Health Research Involving Human Subjects and Clinical Trials Act enacted from April 2018 in Japan. All patients provided their written informed consent prior to enrolment.

### Treatment and follow-up

TME with LLND was performed as previously described (2,9). Combined resection of the surrounding organs or tissues or total pelvic exenteration was permitted to obtain R0 resection. Open surgery alone was planned at the beginning of the trial, and laparoscopic surgery was additionally permitted in May 2018. For surgical quality control and assurance, intraoperative photographs were taken. In the postoperative arm, postoperative chemotherapy with mFOLFOX6 was started 4–8 weeks after surgery and repeated every 2 weeks for 12 courses for pathological Stage II or III patients. mFOLFOX6 consisted of oxaliplatin (85 mg/m^2^, intravenous) with L-leucovorin (200 mg/m^2^, over 2 hours) followed by 5-fluorouracil (400 mg/m^2^, bolus and 2400 mg/m^2^, continuous infusion over 46 hours). In the perioperative arm, 6 courses of mFOLFOX6 were administered before surgery unless disease progression was observed on thoracic/abdominal/pelvic CT performed in week 2 of course 3. Surgery was performed 2–6 weeks after 6 courses of preoperative chemotherapy. The remaining 6 courses of mFOLFOX6 were initiated 4–8 weeks after surgery for Stage 0–III patients including pathological complete response (pCR). Patients were followed-up every 3 months for the first 3 years, and every 6 months for the next 3 years. Follow-up evaluations included severity of neuropathy, measurement of carcinoembryonic antigen and cancer antigen (CA19-9) as tumor marker tests at each examination, and thoracic/abdominal/pelvic CT at 6-month intervals.

### Outcomes

The primary endpoint was OS, defined as the time from randomization until death from any cause). The secondary endpoints included: progression-free survival (PFS), local progression-free survival, proportion of patients with R0 resection, overall response rate to preoperative chemotherapy (perioperative chemotherapy arm), pCR rate (perioperative chemotherapy arm), incidence of adverse events, incidence of serious adverse events, and the proportions of patients who completed 12 courses of chemotherapy, operative complications, surgery without resection of adjacent organs, anus-preservation, and anus-preservation without stoma. Adverse events and postoperative complications were assessed in accordance with the National Cancer Institute Common Terminology Criteria for Adverse Events (version 4.0).

### Statistical analyses

In phase II part, when proportion of R0 resection in the perioperative arm dropped >10% below that in the postoperative arm, this study would be terminated. If the expected value of the primary endpoint of phase II part was 95% in both groups, 30 patients were required in each group in order to maintain a one-sided alpha of 5%. In phase III part, we estimated that the 5-year OS of the perioperative and postoperative arms would be 60 and 50%, respectively. The required sample size was 326 patients (*n* = 163 per arm) to observe 203 deaths, with a one-sided alpha level of 5% and a power of 70% during 7 years of accrual and 5 years of follow-up. Given that some patients would likely be lost to follow-up, the total target sample size was set at 330 patients. Data from all randomized patients were analyzed for OS, PFS, and local progression-free survival on an intention-to-treat basis. Survival curves were estimated using the Kaplan–Meier method and compared using a log-rank test. Hazard ratios were estimated using a Cox regression model. Continuous data was analyzed by Wilcoxon-rank sum test and categorical data was analyzed by Fisher’s exact test. Adverse events were assessed on a per-protocol basis. All *P* values were two-sided except for primary endpoint. Statistical analyses were performed using the SAS software program (version 9.4).

## Results

This randomized trial was started in 18 May 2015. However, it was terminated early in 27 May 2019 due to poor patient enrolment.

### Trial profile

**Figure 2:**
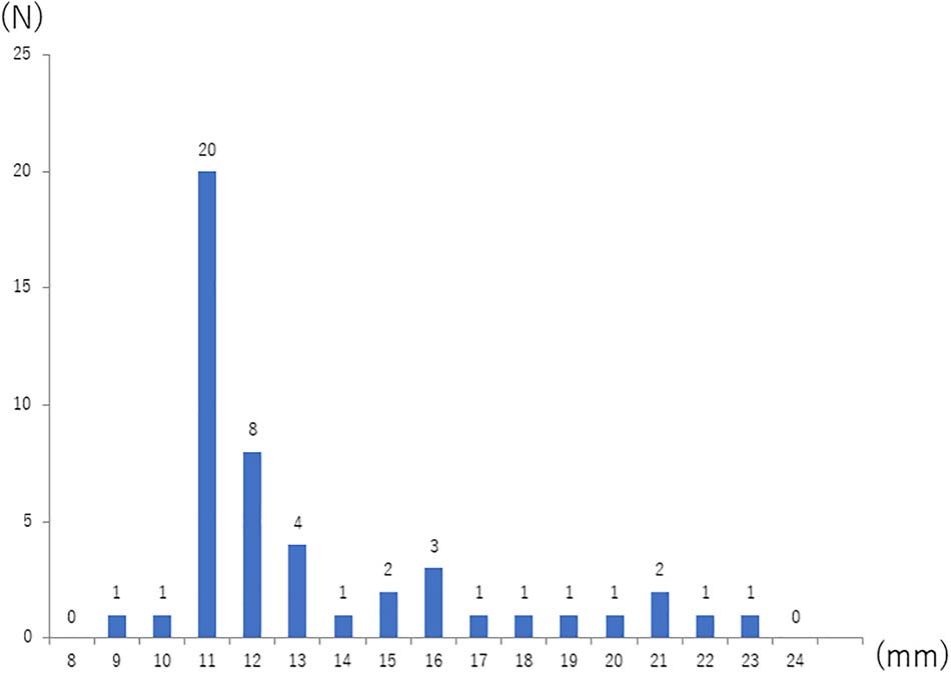
Distribution of the maximal short-axis diameter of the lateral pelvic lymph nodes.

During the corresponding period, 48 patients were enrolled and randomly assigned to the postoperative mFOLFOX6 arm (*n* = 26) or the perioperative mFOLFOX6 arm (*n* = 22) at 18 institutions in Japan (Fig. 1). One patient in the postoperative arm had a lateral pelvic lymph node of 9.0 mm in short axis diameter soon after registration and was judged as ineligible. All other patients had lateral pelvic lymph nodes with a short axis diameter of ≥10 mm. The median short axis diameter of all patients was 11.2 mm (IQR: 10.7–14.9), and the distribution of the sizes is shown in Fig. 2. All 54 clinical LLNMs were located laterally (left 32, right 22) and clustered in the lymph node stations of the obturator nodes (24), distal internal iliac nodes (24), proximal internal iliac nodes (5) and common iliac nodes (1). Two LLNMs were observed in seven patients (left 4, right 3). The baseline characteristics were well balanced between the two arms (Table 1). Among 48 patients with clinical LLNM, 42 had Rb tumors (tumor center located below the peritoneal reflection), the distance from the lower margin to the anal verge was 4.0 cm (IQR: 3.0–5.0), and 4 had a depth of cT2, 28 cT3, 11 cT4a and 5 cT4b.

**Table 1:** Patient characteristics

Characteristics	Postoperative arm (*n* = 26)	Perioperative arm (*n* = 22)	Total (*n* = 48)
Age (years)			
Median	59.5	64.5	61.5
Range	30–71	41–74	30–74
Sex			
Male	19	16	35
Female	7	6	13
Main location			
Ra	2	4	6
Rb	24	18	42
Depth of tumor			
cT2	3	1	4
cT3	14	14	28
cT4a	5	6	11
cT4b	4	1	5
ECOG performance status			
0	25	21	46
1	1	1	2

Ra: Tumor center located above the peritoneal reflection; Rb: Tumor center located below the peritoneal reflection.

### Preoperative chemotherapy

In the perioperative arm, 19 of 22 patents received preoperative chemotherapy. Three patients did not receive preoperative chemotherapy due to patient refusal. One patient died of suicide for unknown reason at the sixth course of preoperative mFOLFOX6. Seventeen of 19 patients completed 6 courses of preoperative mFOLFOX6. Neutropenia was the most frequent grade 3/4 adverse event (*n* = 7, 36.8%) followed by leucopenia (*n* = 2, 10.5%).

### Surgical operation and complications

Progressive disease was not observed on CT in the perioperative arm. TME with LLND was performed in 43 patients (open surgery, *n* = 41; laparoscopic surgery, *n* = 2) according to the protocol. The median operation time was 515 minutes (IQR: 417–645) in the postoperative arm and 491 minutes (IQR: 442–570) in the perioperative arm. The median intraoperative bleeding was 870 ml (IQR: 368–1315) in the postoperative arm and 854 ml (IQR: 550–1240) in the perioperative arm. The procedures of the postoperative (*n* = 25) and perioperative (*n* = 18) arms, respectively, included low anterior resection (*n* = 11 and 5), intersphincteric resection (*n* = 3 and 6), and abdominoperineal resection (*n* = 11 and 7). These procedures were combined surrounding organ resection in seven patients in the postoperative arm and three patients in the perioperative arm. A permanent stoma was created in 11 (44%) patients and 7 (39%) patients in the postoperative and perioperative arms, respectively (*P = 0.765*). Table 2 shows the grade 3 postoperative complications along with laboratory findings. No grade 4 complications were observed. The overall early-stage grade 3 complications, including postoperative bleeding, abdominal infection, pelvic infection, wound infection, ileus and urinary obstruction, were more frequently observed in the perioperative arm than in the postoperative arm (50.0 vs. 12.0%). The postoperative hospital stay was 20 days (IQR: 16–27) and 22 days (IQR: 15–30) in the postoperative and perioperative arms, respectively. No reoperation was performed and no mortality was observed within 30 days after surgery.

**Table 2:** Surgical complications

Complications of Grade 3	Postoperative arm (*n* = 25)	Perioperative arm (*n* = 18)
Intraoperative complications		
Intraoperative hemorrhage	1 (4.0)	0
Postoperative early-stage complications		
Anemia	4 (16.0)	0
Hypoalbuminemia	4 (16.0)	2 (11.1)
Total bilirubin	0	1 (5.6)
AST	0	1 (5.6)
ALT	1 (4.0)	1 (5.6)
Postoperative bleeding	0	1 (5.6)
Abdominal infection	0	1 (5.6)
Pelvic infection	3 (12.0)	3 (16.7)
Wound infection	0	1 (5.6)
Ileus	0	3 (16.7)
Urinary obstruction	0	2 (11.1)
Overall early-stage surgical complications	3 (12.0)	9 (50.0)
Postoperative late-stage complications		
Diarrhea	0	1 (5.6)
Urinary obstruction	0	1 (5.6)

Early-stage: until the first discharge from hospital; late-stage: after the first discharge from hospital; Data are shown as *n* (%). Surgical complications include postoperative bleeding, abdominal infection, pelvic infection, wound infection, ileus, urinary obstruction.

### Pathology

In this study, we defined suspected LLNM as nodes with a short-axis diameter of ≥10 mm on MRI or CT. Pathological examination revealed microscopic LLNM in 20 of 25 (80%) patients who received upfront surgery. As shown in Table 3, in the perioperative arm, the overall response, downstaging (ypStage 0 to 1), and pCR rates were 50.0 (11/22), 22 (4/18) and 9.1% (2/22), respectively. The median long and short diameters of the main tumor were significantly smaller in the perioperative arm (3.25 and 2.5 cm) than in the postoperative arm (6.0 and 4.0 cm). The number of median dissected lymph nodes was decreased from 49.0 (IQR: 44.0–60.0) in the postoperative arm to 40.5 (IQR: 29.0–53.0) in the perioperative arm (*P =* 0.022). The number of metastatic lymph nodes was also decreased from 3.0 (IQR: 2.0–10.0) to 2.0 (IQR: 0.0–6.0) (*P* = 0.239). R0 resection was performed for 42 of 43 patients (one patient in the postoperative arm received R1 resection).

**Table 3:** Representative outcomes of surgery and chemotherapy

Outcomes	Analyzed population	Postoperative arm	Perioperative arm
Proportion of patients with R0 resection	All randomized	92.3% (24/26)	81.8% (18/22)
	Operated	96.0% (24/25)	100% (18/18)
Proportion of patients who completed postoperative chemotherapy	Postoperative chemotherapy	63.2% (12/19)	94.4% (17/18)
Overall response rate of preoperative chemotherapy	Perioperative arm		50.0% (11/22)
Pathological complete response rate	Perioperative arm		9.1% (2/22)
Number of serious adverse events	All randomized	1 (TRD)	1 (death of suicide)
Proportion of preservation of adjacent organs	Operated	72.0% (18/25)	83.3% (15/18)
Proportion of anus- preservation	Operated	56.0% (14/25)	61.1% (11/18)

TRD: treatment-related death.

### Postoperative chemotherapy

As shown in Fig. 1, among 25 patients who underwent surgery in the postoperative arm, two patients with pathological Stage I received no postoperative chemotherapy according to the study protocol. Two patients refused postoperative chemotherapy and one patient who had undergone R1 resection and thereafter developed postoperative complications could not receive chemotherapy. In one patient, the protocol treatment was stopped because of the early termination of the trial. Nineteen patients received postoperative chemotherapy after surgery. Two patients refused to continue chemotherapy due to adverse events. One patient discontinued chemotherapy due to grade 3 pneumonia. Three patients stopped treatment because of the early termination of this trial. In the perioperative arm, 17 of 18 patients completed postoperative chemotherapy. The remaining patient developed peritoneal dissemination and discontinued the protocol treatment. As shown in Table 4, the most common grade 3/4 adverse events in the postoperative and perioperative arms, respectively, were neutropenia (21.1 and 27.8%), diarrhea (15.8 and 0%), sensory neuropathy (10.5 and 11.1%) and leucopenia (0 and 11.1%). The frequency of sensory neuropathy due to oxaliplatin was similar between the arms. One treatment-related death occurred due to sepsis from pelvic infection 154 days after surgery in the postoperative arm. As shown in Table 5, a higher dose of bolus 5-fluorouracil, infusional 5-fluorouracil and oxaliplatin were delivered in the perioperative arm. Among them, oxaliplatin tended to be delivered at a higher dose in the perioperative arm (*P* = 0.085).

**Table 4:** Adverse events of postoperative chemotherapy

Adverse effects of Grade 3/4^a^	Postoperative arm (*n* = 19)	Perioperative arm (*n* = 18)
Leucopenia	0	2 (11.1)
Neutropenia	4 (21.1)	5 (27.8)
ALT	1 (5.3)	0
Diarrhea	3 (15.8)	0
Nausea	1 (5.3)	1 (5.6)
Vomiting	1 (5.3)	1 (5.6)
Allergic reaction	1 (5.3)	1 (5.6)
Anaphylaxis	1 (5.3)	0
Pneumonitis	1 (5.3)	0
Pelvic infection	1 (5.3)	0
Sensory neuropathy	2 (10.5)	2 (11.1)

Data are shown as (%).

^a^Grade 4 was observed only in one patient with neutropenia in the perioperative arm.

**Table 5:** Total administered dose of each drug per body surface area at baseline (mg/m^2^)

Drug	Postoperative arm (*n* = 19)			Perioperative arm (*n* = 19)			*P* value
	25% percentile	Median	75% percentile	25% percentile	Median	75% percentile	
Bolus 5-fluorouracil	3036.3	4105.7	4640.1	3906.2	4350.8	4662.3	0.209
Infusional 5-fluorouracil	21600.0	26390.1	28441.6	25602.2	27762.4	28648.9	0.231
L-leucovorine	1901.1	2295.9	2355.6	2260.5	2287.3	2337.7	0.838
Oxaliplatin	499.4	760.6	893.6	715.1	896.5	972.4	0.085

### The prognosis

**Figure 3:**
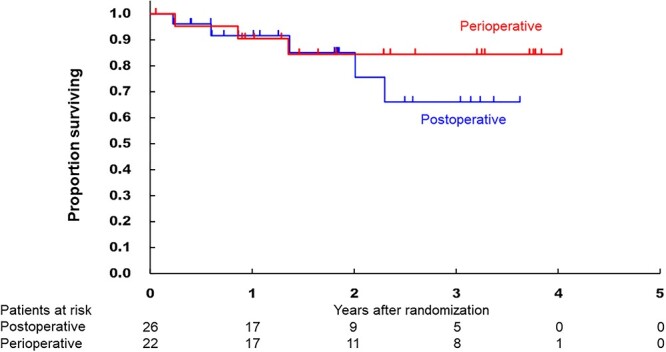
Kaplan–Meier curves of the overall survival in the intention-to-treat populations according to the treatment arm.

**Figure 4:**
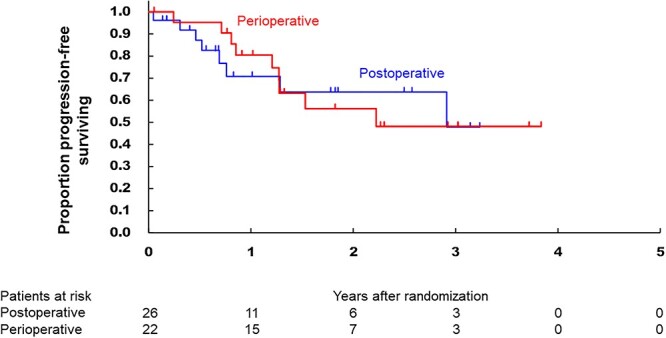
Kaplan–Meier curves of the progression-free survival in the intention-to-treat populations according to the treatment arm.

**Figure 5:**
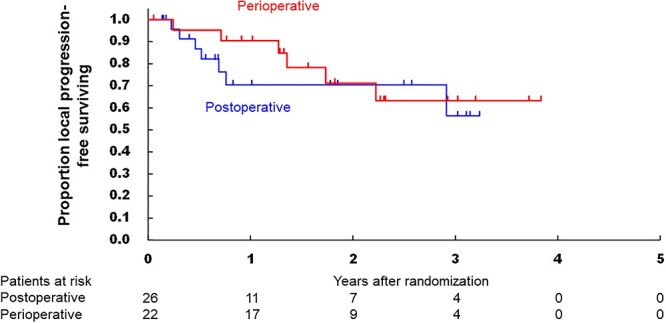
Kaplan–Meier curves of the local progression-free survival in the intention-to-treat populations according to the treatment arm.

All randomized patients were included in the efficacy analysis. When the database was fixed, the median follow-up period was 21.8 months (IQR: 10.6–37.1). Figure 3 shows the OS of the two arms, which were not significantly different (HR, 0.58 95% CI [0.14–2.45], one-sided *P* = 0.228). The 3-year OS in the postoperative and perioperative arms were 66.1% (95% CI 33.9–85.4) and 84.4% (95% CI 58.7–94.8), respectively. The subgroup analysis of OS according to baseline characteristics showed there were no factors being associated with the treatment effect (data not shown). Figure 4 shows the PFS curves, in which the 3-year PFS in the postoperative and perioperative arms were 47.8% (95% CI 16.2–74.1) and 48.1% (95% CI 22.8–69.7) (HR, 0.96 95% CI [0.37–2.49]), respectively. In Fig. 5, 3-year local progression-free survival were 56.3% (95% CI 23.9–79.5) and 63.3% (95% CI 34.6–82.1) (HR, 0.73 95% CI [0.24–2.19]). The recurrent sites did not significantly differ between the two arms (Table 6).

**Table 6:** Patterns of recurrence

Recurrence site	Postoperative arm (*n* = 26)	Perioperative arm (*n* = 22)
Total recurrent patients	8	8
Lung	3	4
Liver	1	2
Local		
Anastomosis	0	1
Intra-pelvis	6	3
(Central pelvis)	(4)	(1)
(Lateral pelvis)	(4)	(2)
Perineum	0	1
Others	3	3

There are some overlapping data.

## Discussion

To our knowledge, this is the first randomized controlled trial to investigate the superiority of perioperative chemotherapy to postoperative chemotherapy in terms of OS in high-risk rectal cancer with LLNM (9). We hypothesized that preoperative chemotherapy might ameliorate compliance with chemotherapy and consequently improve OS in the perioperative arm.

Higher early-stage grade 3 postoperative surgical complications in the preoperative arm, which is not negligible, may be due to some adverse effects of preoperative chemotherapy. However, no grade 4 complications and no reoperation were observed. More patients are therefore needed to reach a conclusion concerning this issue. At present, we believe that perioperative chemotherapy was relatively safe and well-tolerated with only one lethal event unrelated to the protocol treatment. The proportion of patients who completed postoperative chemotherapy was higher in the perioperative arm. The total dose of mFOLFOX6 was delivered at a higher dose in the perioperative arm. As for the efficacy, the 3-year OS tended to be higher in the perioperative arm (84.4 vs. 66.1%) without statistical significance because this trial was terminated early with poor patient accrual. The administration of a far greater dose of mFOLFOX6 may have been needed to positively influence OS in the perioperative chemotherapy arm in comparison to the postoperative arm. Although we initially considered 6 courses of preoperative mFOLFOX6 followed by 12 courses of postoperative mFOLFOX6 for the perioperative arm, the administration of 18 courses of mFOLFOX6 prompted concerns about severe neurotoxicity (9). Preoperative triplet chemotherapy with FOLFOXIRI instead of mFOLFOX6 may be a candidate because Glynme-Jones et al. reported that a FOLFOXIRI plus bevacizumab group showed better OS than a FOLFOX plus bevacizumab group in a randomized non-comparative phase II study of preoperative chemotherapy for rectal cancer (17).

Most reports on preoperative chemotherapy alone for rectal cancer were small-sized, single-armed, included heterogeneous chemotherapy regimens, and were not phase III studies (18). However, Deng et al. reported the results of the FOWARK phase III trial comparing three treatment arms with TME alone for Stage II/III LARC: preoperative 5-fluorouracil-Radiotherapy plus postoperative 5-fluorouracil, preoperative mFOLFOX6-Radiotherapy plus postoperative mFOLFOX6, and perioperative mFOLFOX6 alone (19). The perioperative mFOLFOX6 alone arm had a lower pCR rate but similar 3-year OS to the 5-fluorouracil-radiotherapy arm, with less toxicity and fewer postoperative complications. In comparison to their perioperative chemotherapy arm, the present perioperative chemotherapy arm showed a higher pCR rate (9.1% vs. 6.6%) and a proportion of R0 resection (100% vs. 89.4%), however, lower 3-year OS (84.4% vs. 90.7%). The higher proportion of R0 resection may be because TME with LLND is more aggressive than TME alone. The lower 3-year OS may be due to the higher malignant potential of rectal cancer with LLNM ≥10 mm as the short-axis diameter was reported to be significantly associated with the local recurrence-free survival, RFS, and OS (20). Schrag et al. have been conducting the randomized controlled PROSPECT trial, comparing standard preoperative CRT and preoperative FOLFOX for relatively low-grade and highly located rectal cancer (21). The long-term oncological outcomes are awaited. The two abovementioned trials differ from the present trial in that they can use additional radiation or selective chemoradiation for patients with LARC, even in the FOLFOX arm. Regarding local recurrence, Kim et al. reported that the 5-year local recurrence-free survival was 40.1% in the patients with clinical LLNM ≥10 mm (20), which was comparable to our data (Fig. 5). Preoperative CRT followed by TME with LLND may be one of the most powerful tools for local control. Ishihara et al. reported in a retrospective study that the 5-year local recurrence rate was 0% after performing the strategy for lower rectal cancer with clinical LLNM ≥8 mm (22).

In Western countries, where preoperative CRT followed by TME is a standard treatment for rectal cancer, a new treatment called ‘Total Neoadjuvant Treatment’ has recently been reported in randomized controlled trials (23,24). The RAPIDO trial introduced preoperative consolidation CAPOX/FOLFOX and improved disease-free survival (23). The PRODIGE23 trial introduced preoperative induction FOLFIRINOX and improved disease-related treatment failure (24). Regrettably, neither treatment significantly ameliorated OS. However, additional preoperative intensive chemotherapy to CRT and delayed surgery is highly expected to improve survival, unlike standard CRT alone. Neoadjuvant FOLFIRINOX or FOLFOXIRI may play a crucial role in the treatment of LARC with or without CRT.

The present study is associated with some limitations, mainly because it was terminated early due to poor accrual (48 out of 330 patients). Several reasons are proposed for the poor accrual. First, the incidence of pathological LLNM in patients with T3 or T4 lower rectal cancer is reported to be 18.1% (25). However, the number of resectable patients with lateral pelvic lymph node with a short-axis diameter of ≥10 mm was far less than expected based on the JCOG Colorectal Cancer Study Group questionnaire that was conducted when preparing the protocol. Second, open surgery alone was permitted at the beginning of the study in May 2015 to ensure surgical security for challenging TME with LLND, including total pelvic exenteration, for LARC. Laparoscopic surgery was permitted in May 2018 to promote patient recruitment with its prevalence, however, only two patients underwent laparoscopic surgery before May 2019.

In conclusion, although the sample size was small due to early termination, our findings suggest that perioperative mFOLFOX6 may be an insufficient treatment with anti-cancer efficacy to improve the survival of lower rectal cancer with lateral pelvic lymph node metastasis. More intensive treatment, such as additional chemoradiation or the FOLFIRINOX regimen, might be needed.
